# Sex differences in gene expression related to antipsychotic induced weight gain

**DOI:** 10.1371/journal.pone.0215477

**Published:** 2019-04-15

**Authors:** Jesus Sainz, Carlos Prieto, Benedicto Crespo-Facorro

**Affiliations:** 1 Spanish National Research Council (CSIC), Institute of Biomedicine and Biotechnology of Cantabria (IBBTEC), Santander, Spain; 2 Bioinformatics Service, Nucleus, University of Salamanca (USAL), Salamanca, Spain; 3 University Hospital Marqués de Valdecilla, IDIVAL, Department of Psychiatry, School of Medicine, University of Cantabria, Santander, Spain; 4 CIBERSAM, Centro Investigación Biomédica en Red Salud Mental, Santander, Spain; 5 University Hospital Virgen del Rocio, University of Sevilla, Seville, Spain; Maastricht University, NETHERLANDS

## Abstract

Antipsychotics are crucial for the treatment of schizophrenia and contribute to weight gain in psychosis, particularly during early phases. Antipsychotic Induced Weight Gain (AIWG) might contribute to reduce the quality of life, drug compliance and to increase mortality. To characterize sex differences of gene expression related to AIWG, we sequenced total mRNA from blood samples of schizophrenia patients, before and after 3 months of antipsychotic-treatment. We analyzed schizophrenia patients according to their sex (38 males and 39 females) and their BMI increase after medication, characterizing the differential gene expression before and after medication. Individuals in each group were categorized in patients who gain weight and those whose do not gain weight. The “weight gain” groups included patients with an increase of body mass index (BMI) > 1.0 points (27 males and 23 females with a median BMI increase of 2.68 and 2.32 respectively). The “no weight gain” groups included patients with a change of BMI between < 1.0 and > -1.0 points (11 males and 16 females with a median BMI increase of 0.21 and 0.16 respectively). The males had 331 genes with significant differential expression in the weight gain group and 24 genes in the no weight gain group. The females had 119 genes with significant differential expression in the weight gain group and 75 genes in the no weight gain group. Both weight gain groups were significantly enriched with “obesity” genes (Fisher; p = 1.1E-09 and p = 0.0001 respectively), according to the Gene Reference into Function (GeneRIF) database.In conclusion, we characterized genes with differential expression associated to AIWG that are specific to males, to females and common to both sexes. These genes are good candidates to depict the biological processes involved in AIWG and provide additional evidence of the genetic links between weight gain and the immune system.

## Introduction

**Fig 1:**
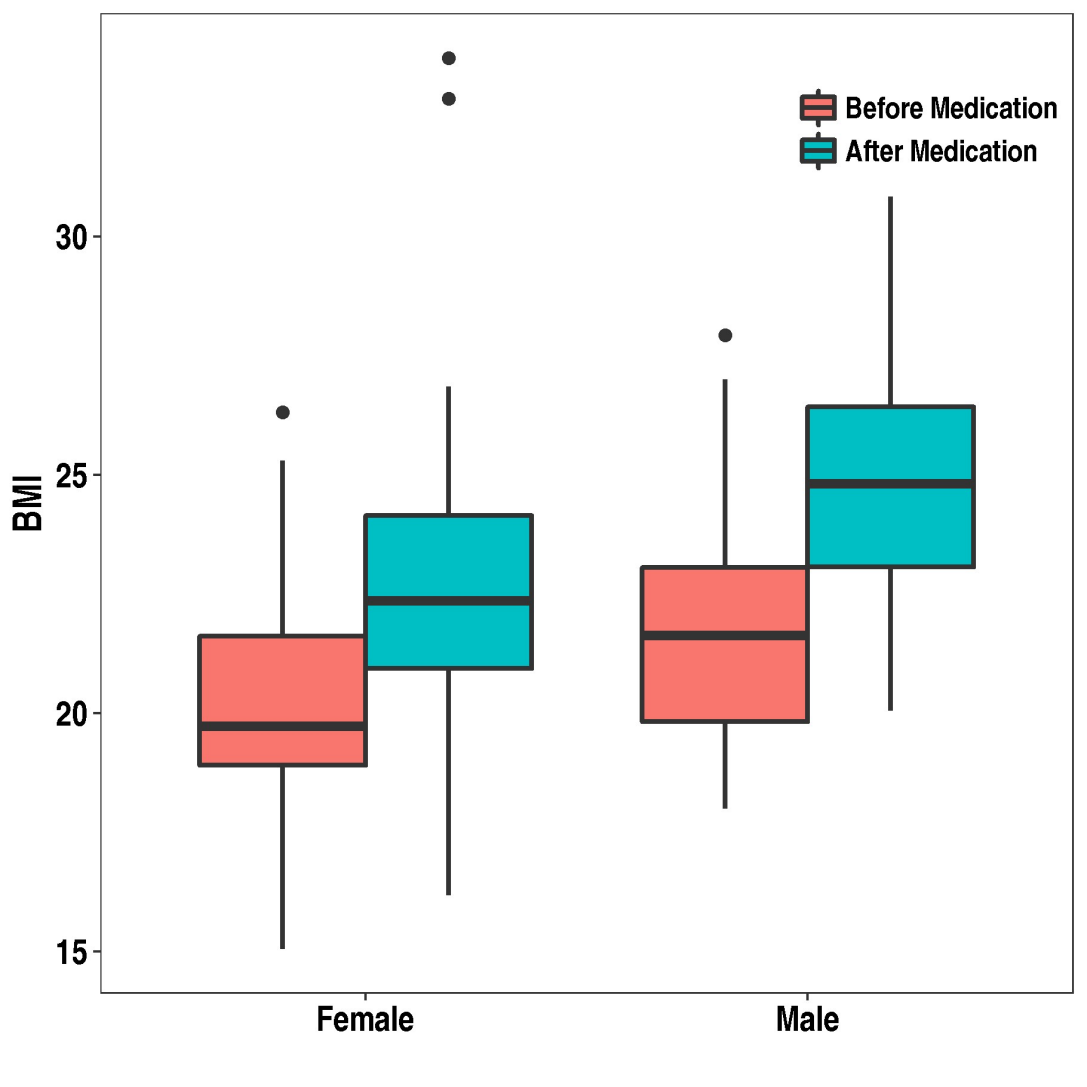
Whisker plot of body mass index (BMI) data in the male and female weight gain groups.

Antipsychotic medications are the keystone for the treatment of schizophrenia and other psychotic disorders (including schizoaffective disorder, delusional disorder and bipolar affective disorder)[1], although they are also extensively used in several other disorders such as personality disorders, dementia and autism[2–4]. The clinical differences between antipsychotic drugs are mainly in the areas of safety and tolerability. The second-generation antipsychotics (SGAs) as a group are recommended, due to a lesser association with extrapyramidal symptoms and hyperprolactinemia, by most guidelines as first-line therapy for schizophrenia[5]. Although several other factors (i.e.; genetic susceptibility, sedentary lifestyle and unhealthy food habits) may account for weight gaining in patients with psychosis, antipsychotic treatment (especially SGAs) is considered a significant contributor to weight gain, in particular during early phases of psychosis[6–8]. In consequence, antipsychotic-induced weight gain (AIWG) is a major management problem for clinicians, given that obesity and overweight have a strong negative impact in health[9]. Weight gain is a risk factor for diabetes type II and can lead to metabolic syndrome, a severe condition with serious implications for survival[10]. Thus, the consequences of a long-term treatment with antipsychotics comprise not only overweight and obesity, but also metabolic disturbances and cardiovascular disease[11, 12], although cardiovascular mortality in schizophrenia is attributable to other factors than antipsychotic treatment when used in adequate dosages[13]. The reduced life expectancy for schizophrenic patients is attributable in a great extent to a higher rate of cardiovascular disease[14–17]. There are great differences in obesity according to sex. Overall, more women are obese than men, yet, in developed countries, more men are overweight than women[18]. The reasons of gender differences in obesity and the molecular mechanisms behind AIWG are not well known. Given that that AIWG has a strong genetic component[19], we analyzed the transcriptome expression changes related to weight gain caused by antipsychotics. To obtain information about the genetics behind the sex differences, we analyzed independently males and females comparing individuals with weight gain and without it. We sequenced the blood transcriptome of male and female cohorts of first episode schizophrenia patients before (antipsychotic naïve) and after three months of treatment with antipsychotics (Table 1). The “weight gain” cohorts included 27 patients in the male group and 23 patients in the female group that gained more than 1.0 points of BMI after the treatment (Fig 1). The “no weight gain” groups included 11 males and 16 females with a change on BMI after medication lower than 1.0 points and higher than -1.0 points. The transcriptomes of the four different groups were analyzed independently to define genes with significant differential expression before and after medication in the male and female weight and no weight gain groups using the program Deseq[20].

**Table 1:** Anthropometric and metabolic values in the groups studied.

	Weight Gain		No weight gain	
Median	Male (N = 27)	Female (N = 23)	Male (N = 11)	Female (N = 16)
Age	24,8	26,8	35,7	41,0
BMI before medication	21,6	19,7	26,8	20,4
BMI after medication	24,8	22,3	26,2	20,6
BMI increase	2,68	2,32	0,21	0,16
Triglycerides before medication (mmol/L)	85	61	83	74
Triglycerides after medication (mmol/L)	107	87	98	92
Cholesterol before medication (mg/dl)	154	167	198	173
Cholesterol after medication (mg/dl)	166	192	195	183

## Materials and methods

### Study setting and subjects

The cohort analyzed in this study was obtained at the University Hospital Marques de Valdecilla (Cantabria, Spain). Conforming to international standards for research ethics, this study was approved by the Cantabria Ethics Institutional Review Board (IRB). Patients meeting inclusion criteria and their families provided written informed consent to be included in the study. The biological samples of patients were provided by the Valdecilla biobank (Santander). All methods were performed in accordance with the relevant guidelines and regulations. Before giving informed consent, all patients were evaluated by a clinician to assess their competency (according to their capacity of understanding, reasoning, and expression of a choice). After informed consent was signed, patients were included in a prospective, randomized, flexible-dose, open-label study.

All patients included in the study met the following criteria: 1) 15–60 years old; 2) living in the catchment area (Cantabria); 3) experiencing a first episode of psychosis; 4) having received no prior treatment with antipsychotic medication; 5) DSM-IV criteria for schizophrenia, schizophreniform disorder, schizoaffective disorder, or brief psychotic disorder. Patients were excluded from the study for any of the following reasons: 1) meeting DSM-IV criteria for drug dependence, 2) meeting DSM-IV criteria for mental retardation, 3) having a history of neurological disease or head injury. The diagnoses were confirmed, using the Structured Clinical Interview for DSM-IV (SCID–I), by an experienced psychiatrist after 6 months of the baseline visit. Our operational definition for a “first episode of psychosis” includes individuals with a non-affective psychosis who have not previously received antipsychotic treatment regardless the duration of psychosis.Only individuals who gave written consent to their participation in the program, fulfilled inclusion criteria at 6 months, and had mRNA samples available at baseline and at 3 months were finally included in the present study (S1 Fig.). No statistically significant differences were found in clinical and sociodemographic characteristics at baseline between those patients included (N = 77) and no included (N = 98) in final analyses (all ps > 0.05), except for CGI and BPRS (S1 Table).

Anthropometric and metabolic values (age, body mass index, triglycerides and cholesterol) were collected from patients before and after medication in the groups studied. (Table 1).

### Sample and study design

We selected two groups of patients to sequence their mRNA from blood samples before and after 3 months of treatment with antipsychotics. We defined the “weight gain” groups by an increase of BMI > 1.0 points; and the “no weight gain” group by a change of BMI between < 1.0 and > -1.0 points. The “weight gain” cohorts included 27 patients in the male group and 23 patients in the female group. The “no weight gain” groups included 11 males and 16 females.

### Laboratory assessments

Blood samples were assessed for biochemical and hematological parameters. Blood samples were obtained from fasting subjects from 8:00 to 10:00 a.m. by the same staff and in the same setting. A detailed description of methodology followed to assess biochemical variables is available under request. None of the patients had a chronic inflammation or infection, or were taking medication that could influence the results of blood tests.

### RNA extraction

Total RNA was extracted from blood using the Tempus Blood RNA Tube and Tempus Spin RNA Isolation Kit (Applied Biosystems, Foster City, CA, USA) following the manufacturer protocols. To define expression profiles, a key factor is that the RNA is intact. To select only high-quality RNA, the RNA Integrity Number (RIN) was characterized with a Bioanalyzer (Agilent Technologies, Santa Clara, CA, USA) and samples with a RIN of at least 7.4 were selected. We selected this cut-off because it ensures a minimum of quality regarding mRNA integrity. The majority of the selected samples have RINs that range from 8 to 10 with an average of 9.1.

### RNA Next generation sequencing

Total RNA was extracted from peripheral blood of each individual. The mRNA obtained from blood was sequenced at the Centro Nacional de Análisis Genómico (CNAG) using Illumina HiSeq instruments (San Diego, CA, USA). The mRNA was isolated from the total RNA and was fragmented once transformed into cDNA. Fragments of 300 bp on average were selected to construct the cDNA libraries for sequencing by the “Centro Nacional de Análisis Genómico” (CNAG). These libraries did not included ncRNA, only mRNA. Pair-end sequences of 70 nucleotides for each end were produced.

### Alignment of reads to the human genome reference

Alignment of the reads was performed in an SLURM HPC server running Tophat 2.0.6 with default options[21]. Tophat aligns RNA-Seq reads to genomes using the Bowtie 2.0.2 alignment program[22], and then analyzes the mapping results to identify splice junctions between exons.

### Differential expression and statistical analyses

Bedtools 2.17.0 (multicov option)[23] was used to count the amount of reads mapped to each gene. The Reference Sequence (RefSeq) gene coordinates were defined using the RefFlat file from the UCSC Genome Bioinformatics Site (as February 28th, 2014). Deseq 1.4 package[20], setting up fit-only as fitting method, was used to test for differential expression using gene-count data. P value was adjusted for multiple testing with the Benjamini-Hochberg procedure, which controls false discovery rate.

Two sided Fisher tests were carried out to identify functional enrichment of biological annotations. Linear regression analyses of expression changes versus BMI changes were performed without obtaining relevant results.

## Results

### Differential gene expression in the male “weight gain” group before and after treatment with antipsychotics

We found 331 genes with significant differential expression before and after medication (Padj value <0.01) (S2 Table).

These genes were significantly enriched for the strings “obesity” or “BMI” according to the scientific literature in the GeneRIF database that provides functional and morbid annotation of genes[24].

We found 42 “obesity” related differential expression genes or 14.4% of the annotated genes while we expect 4.9% (Fisher; p = 1,098E-09). When we analyzed for the string “BMI”, we found 15 genes or 5.1% of the annotated genes while we expect 2.8% (Fisher; p = 0.03).

These differential expression genes were also significantly enriched for “cholesterol” related genes, 43 or 14.8% of the annotated genes while we expect 5.2% (Fisher; 1,776E-09); and for “triglyceride” related genes, 15 or 5.1% of the annotated genes while we expect 1.9% (Fisher; p = 0.0005). Additionally, there was a strong enrichment for diabetes genes: 79 or 27.1% while we expect 10.6% (Fisher; p = 8.28E-15).

### Differential gene expression in the female “weight gain” group before and after treatment with antipsychotics

We found 119 genes with significant differential expression before and after medication (Padj value <0.01) (S3 Table).

These genes were enriched for the string “obesity” and weakly enriched for “BMI” according to the scientific literature in the GeneRIF database[24].

We found 16 “obesity” differential expression genes or 14.7% of the annotated genes while it was expected 4.9% (Fisher; p = 0.0001). When we analyzed for the string “BMI”, we found 7 genes or 6.4% of the annotated genes while it was expected 2.8% (Fisher; p = 0.03). There was also a strong enrichment for diabetes genes: 30 or 27.5% while we expect 10.6% (Fisher; p = 8.09E-07).

The differential expression genes were significantly enriched for “cholesterol” related genes: 16 or 14.7% of the annotated genes while we expect 5.2% (Fisher; p = 0.0002) and for “triglyceride” related genes: 7 or 6.4% of the annotated genes while we expect 1.9% (Fisher; p = 0.005). There was also a strong enrichment for “diabetes” genes: 30 or 27.5% while we expect 10.6% (Fisher; p = 8.09E-07).

### Genes with differential expression specific to the male and female “weight gain” groups only

By subtracting the differential expression genes of the male and female no weight gain groups, and the female weight gain group (S3, S4 and S5 Tables respectively), we found specifically 250 genes with differential expression in the male weight gain group.

By subtracting the differential expression genes of the male and female no weight gain groups, and the male weight gain group (S2, S4 and S5 Tables respectively), we found 37 genes specific to the female weight gain group.

### Common genes to male and female having differential expression only in the “weight gain” groups

We found 47 genes with differential expression common to the male and female weight gain groups (S2 and S3 Tables respectively) and not present in the male nor in the female no weight gain groups (S4 and S5 Tables respectively)

### Pathway analyses

Analyses of several pathway databases (Reactome[25], KEGG[26], WikiPathways[27] and Pathway Interaction Database[28]) revealed that pathways related to the immune system were highly enriched with differential expression genes common to the male and female weight gain groups and not present in the no weight gain groups. The three most significantly gene enriched pathways were all immune system related. Neutrophil Degranulation with 17 genes (Observed 42%, expected 4%; Fisher p = 1.06E-13). Innate Immune System with 19 genes (Observed 47%, expected 11%; Fisher p = 5.6E-09). Immune System with 22 genes (Observed 55%, expected 18%; Fisher p = 1.0E-07).

Using the same type of analyses, we found very similar results for the genes specific to the male and the female weight gain groups respectively.

For the genes specific to the male weight gain group, the three most significantly gene enriched pathways were: Immune System with 71 genes (Observed 40%, expected 18%; Fisher p = 3.3E-12). Innate Immune System with 50 genes (Observed 28%, expected 11%; Fisher p = 1.7E-10). Neutrophil Degranulation with 27 genes (Observed 15%, expected 4%; Fisher p = 7.06E-09).

For the genes specific to the female weight gain group, the three most significantly gene enriched pathways were: Interferon alpha/beta signaling with 5 genes (Observed 18%, expected 1%; Fisher p = 7.47E-07). Interferon Signaling with 6 genes (Observed 21%, expected 2%; Fisher p = 6.4E-06). Immune System with 13 genes (Observed 46%, expected 18%; Fisher p = 0.0004).

### Weighted gene co-expression network analysis (WGCNA) and linear regression analyses

In the linear regression analyses of expression changes versus BMI changes, we did not found any significant gene (Padj<0.001) in males and only seven genes in females (S6 Table). We also performed a WGCNA (Weighted Gene Correlation Network Analysis) and resulting modules were correlated with BMI changes. Some of these modules were significantly correlated with BMI changes but we did not find a functional enrichment of BMI related functions (S7 and S8 Tables).

## Discussion

It is well known that antipsychotics induce weight gain[29] and that genetic factors play a major role in this weight gain.[7]. Our analysis provides genetic expression data that could clarify the genetic component of the weight gain caused by antipsychotics. It is also well known that adolescent patients gain more weight than older patients[15]. In the cohort analyzed here, the median of age of the male and female weight gain groups had a median of age 10.9 and 14.2 years younger, respectively, than the correspondent no weight gain groups (Table 1), consistently with the literature. One hypothesis is that weight gain is more marked and frequent in the younger patients due to less prior antipsychotic exposure compared to the older patients[30]. However, this hypothesis could not explain the differences observed in our cohort given that all are naïve patients. Other explanation is that the younger patients are simply more sensitive to the weight gain caused by antipsychotics[31], though this is not supported by any molecular mechanism underlying it. Here we provide gene functional data that could explain partially the molecular basis of the differences in AIWG between young and adult.

It also has been reported sex differences in the schizophrenia age of onset, being the males age of onset earlier than the females[32]. We observed also in our cohort that males are younger than females (2.0 and 5.3 years younger in the weight and no weight gain groups respectively; Table 1). These sex differences in age could influence the expression profiles we characterized. However, the genes that are specific of the weight gain groups, even if their expression was age-related, they still could explain the molecular mechanisms of obesity given that these changes are associated to antipsychotics intake and to weight gain.

In the male weight gain group, we found 331 genes with differential expression after medication with antipsychotics. Out of the 331 genes, there are 310 genes with expression not altered by medication in the male no weight gain group. After similar analysis for the female groups, we find 94 genes out of the 119 genes with differential expression in the female weight gain group but not in the female no weight gain group.

Among the genes appearing only in the male and female weight gain groups, 47 are common to male and female, representing 50% of the female 94 female weight gain specific genes.

In males, there are 240 genes having altered expression specific to the weight gain group. These 240 male specific genes have no differential expression in any of the other analyses performed (male no weight gain, female weight gain and female no weight gain groups).

In females, we find 37 genes with altered expression specific to the female weight gain group. The expression of these genes was not altered after medication in any of the other three analyzed groups.

Out of the 240 genes with differential expression only in the male weight gain group, 40 genes are related to obesity traits and metabolic syndrome according to the GeneRif literature. The top ten with the most significant differential expression are: *H19*[33], *ADM*[34], *CES1*[35], *S100A8*[36], *ACSL1*[37], *C3AR1*[38], *SDC3*[39], *KLF4*[40], *IFI30*[41] and *FABP1*[42]. Among the remaining of the 40 genes, the cholinergic receptor muscarinic 3, or *CHRM3*, has genetics variants that have been associated with BMI after antipsychotic medication[43]. It is interesting to report that two of the remaining genes that do not appear associated to obesity traits in GeneRif are indeed involved in leptin pathways: *BCL2L1*, *BCL2* like 1, part of the Leptin signaling pathway (WikiPathways); and *NRG1*, neuregulin 1, part of the Signaling by Leptin pathway (Reactome) and also associated to schizophrenia[44].

In females, we find four genes related to obesity traits and metabolic syndrome out of the 37 genes with differential expression specific to the female weight gain group. These genes are *CEACAM1*[45], *SDC1*[46], *PLA2G2A*[47] and *HP*[48].

Out of the 47 common genes to male and female specific to the weight gain groups, 11 relate to obesity traits and metabolic syndrome according to previous studies: *LTF*[49], *OLFM4*[50], *LCN2*[51], *OLR1*[52], *MMP8*[53], *PDK4*[54], *RNASE3*[55], *APOA4*[56], *CHIT1*[57], *GPER1*[58] and *CPT1A*[59]. The gene *OLAH*, oleoyl-ACP hydrolase, has not been reported as obesity-trait related in GeneRif but is included in several Reactome lipid-related pathways: Metabolism of lipids and lipoproteins, Fatty acid, triacylglycerol, and ketone body metabolism, Triglyceride Biosynthesis and Fatty Acyl-CoA Biosynthesis.

When we search for “obesity” in the GeneRIF database, we find a significant enrichment of genes with altered expression after antipsychotic medication in the male and female weight gain groups (Fisher; p = 1.1E-09 and p = 0.0001 respectively). Regarding the remaining genes not published as related to obesity traits, is likely to include genes with a relation still unknown to obesity traits. The pathways that some of these genes are included point in that direction.

Pathway analyses of the genes with altered expression specific to the weight gain groups (common to male and female and specific to male or to female) indicate a consistent enrichment of immune system pathways. These observations support a relationship of weight gain and the immune system, as it has been previously suggested[60–62].

We observe a higher enrichment of obesity genes with expression altered by antipsychotics in the males than in the females. A possible explanation for this observation could be that the male groups are younger than the female groups. This is consistent with the studies that report that young people are more sensitive to AIWG. However, we cannot exclude other factors such as BMI before medication; males have a median of 1.9 and 6.4 points of higher BMI than the females in the weight gain and no weight gain groups respectively (Fig 1 and Table 1). In conclusion, our study provides expression data of a list of genes that we believe are good candidates to be involved in biological processes associated with AIWG in the patients medicated for psychosis, depression and other diseases. Logically, these genes could be also involved in the molecular mechanisms that lead to obesity traits in the general population. Also provides additional evidence of the genetic links between weight gain and the immune system. Finally, we expect that this study could facilitate creating predictive tools of weight gain and in turns tailoring specific interventions according to individual risks. The data obtained is a first step to generate a tool for clinicians to predict personalized AIWG for each antipsychotic available. Consequently, more studies would be needed to analyze the AIWG for each antipsychotic and to add data from cohorts from different ethnicities.

## Supporting information

S1 Fig Flow chart of patients. (PDF)

S1 Table Comparison of clinical and sociodemographic characteristic between included (N = 77) and no included patients (N = 98). (XLSX)

S2 Table Differential expression in the male weight gain group before and after 3 months of antipsychotic medication. (XLSX)

S3 Table Differential expression in the female weight gain group before and after 3 months of antipsychotic medication. (XLSX)

S4 Table Differential expression in the male no weight gain group before and after 3 months of antipsychotic medication. (XLSX)

S5 Table Differential expression in the female no weight gain group before and after 3 months of antipsychotic medication. (XLSX)

S6 Table Linear regression analyses of expression changes versus BMI changes in females. (XLSX)

S7 Table WGCNA results with male samples. (XLSX)

S8 Table WGCNA results with female samples. (XLSX)
