# Supplementary figures and images for: Sex differences in gene expression related to antipsychotic induced weight gain
Source: PLoS One. 2019 Apr 15;14(4):e0215477. doi: 10.1371/journal.pone.0215477 (PMC6464344; doi:10.1371/journal.pone.0215477)

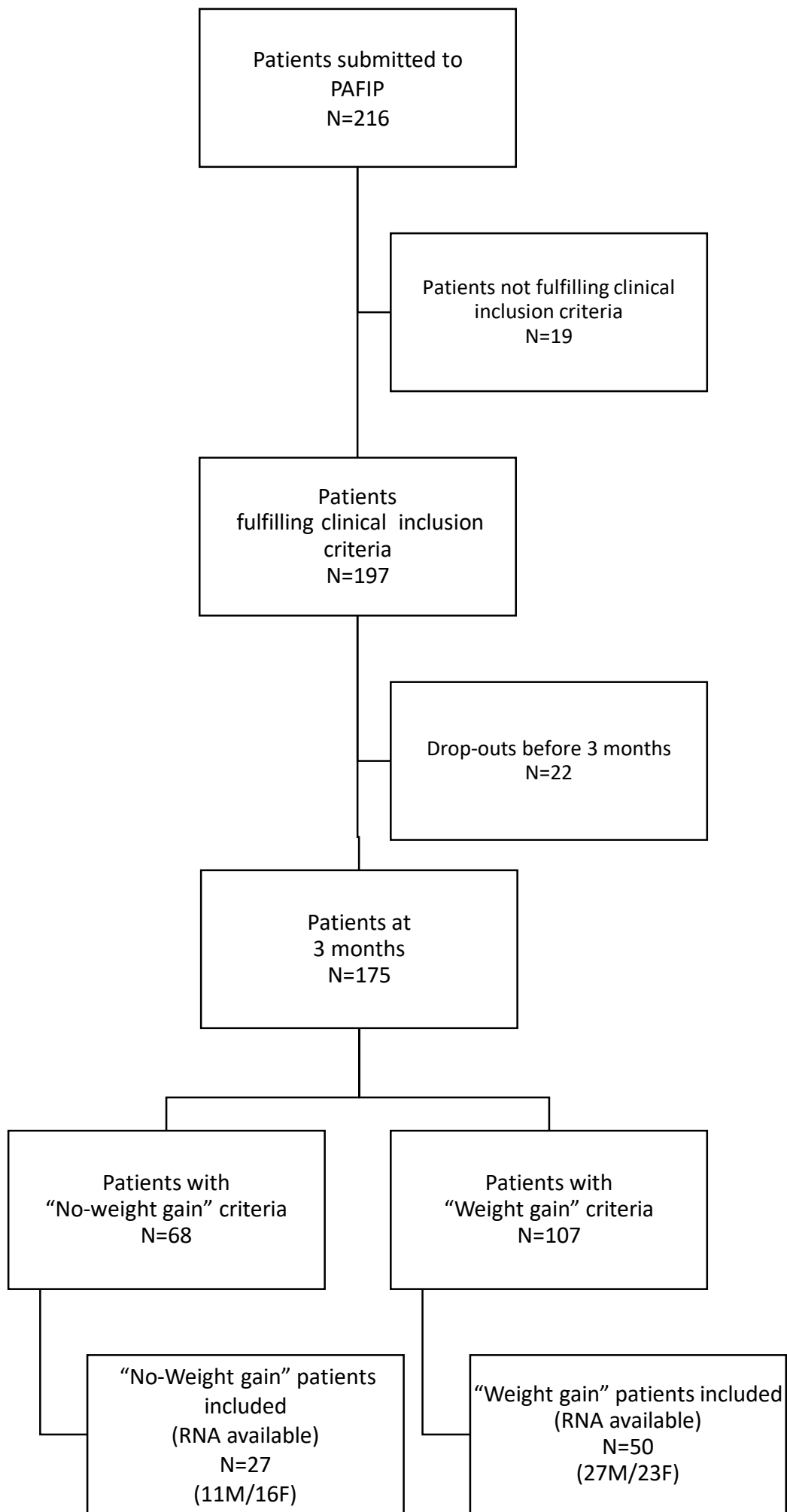

**Abbreviations:**

PAFIP: Programa Asistencial de Fases Iniciales de Psicosis

Supplement: S1 Fig — (PDF) [file pone.0215477.s001.pdf]
